# Framework for interpretation of genetic variations in pancreatitis patients

**DOI:** 10.3389/fphys.2012.00440

**Published:** 2012-12-06

**Authors:** David C. Whitcomb

**Affiliations:** ^1^Division of Gastroenterology, Hepatology, and Nutrition, Department of Medicine, University of Pittsburgh and UPMCPittsburgh, PA, USA; ^2^Department of Human Genetics, University of Pittsburgh and UPMCPittsburgh, PA, USA; ^3^Department of Cell Biology and Molecular Physiology, University of Pittsburgh and UPMCPittsburgh, PA, USA

**Keywords:** pancreatitis, cystic fibrosis, genetics, next generation sequencing, GWAS, systems biology, inflammation

## Abstract

Chronic pancreatitis (CP) is defined by irreversible damage to the pancreas as a result of inflammation-driven pancreatic tissue destruction and fibrosis occurring over many years. The disorder is complex, with multiple etiologies leading to the same tissue pathology, and unpredictable clinical courses with variable pain, exocrine and endocrine organ dysfunction, and cancer. Underlying genetic variants are central CP susceptibility and progression. Three genes, with Mendelian genetic biology (*PRSS1, CFTR*, and *SPINK1*) have been recognized for over a decade, and little progress has been made since then. Furthermore, application of high-throughput genetic techniques, including genome-wide association studies (GWAS) and next generation sequencing (NGS) will provide a large volume of new genetic variants that are associated with CP, but with small independent effect that are impossible to apply in the clinic. The problem of interpretation is using the old framework of the germ theory of disease to understand complex genetic disorders. To understand these variants and translate them into clinically useful information requires a new framework based on modeling and simulation of physiological processes with or without genetic, metabolic and environmental variables considered at the cellular and organ levels, with integration of the immune system, nervous system, tissue injury and repair system, and DNA repair system. The North American Pancreatitis Study 2 (NAPS2) study was designed to capture this type of date and construct a time line to understand and later predict rates of disease progression from the initial symptom to end-stage disease. This effort is needed to target the etiology of pancreatic dysfunction beginning at the first signs of disease and thereby prevent the development of irreversible damage and the complications of CP. The need for a new framework and the rational for implementing it into clinical practice are described.

## Introduction

Classic Mendelian genetics plays a small but significant role in chronic pancreatitis (CP). Three syndromes are well described including autosomal dominant hereditary pancreatitis (HP), autosomal recessive cystic fibrosis (CF), and autosomal recessive familial pancreatitis from homozygous or compound heterozygous *SPINK1* mutations. The biology and pathology of these genes, plus lower risk genes chymotrypsin C (*CTRC*) and calcium sensing receptor (*CASR*), have recently been reviewed (Teich and Mossner, 2008; Chen and Ferec, 2009; Whitcomb, 2010; Larusch and Whitcomb, 2011; Chen and Ferec, 2012). However, these syndromes make up less than 10% of CP cases in most clinical populations.

It is now recognized that non-Mendelian, complex genetic conditions are far more common and therefore of greater relevance. Complex genetics include gene-environment or gene–gene interactions, or more complex combinations and variable interactions. Any one of these disease-associated factors is neither sufficient nor necessary to cause pancreatitis alone, but can contribute to the disease or its complications when present within the right context. Patients with complex diseases rarely come from large families. Rather, the disease appears to be sporadic or occurring in only one or two other family members. Demonstrating the etiologic basis of complex genetic disorders is much more difficult than Mendelian disorders.

The initial excitement of the discovery of three major pancreatitis susceptibility genes between 1996 and 2000 was followed by slow progress in understanding pancreatic genetics, which reflects the depth of the problem and the large number of patients necessary to understand these complex interactions. Two major genome-wide association studies (GWAS) have been completed (one in Germany, and another in the United States) and the results will soon be reported. What is clear is that the results will either apply only to a small subset of patients, or will be important as cofactors or modifiers in more complex interactions. However, what is needed is a new framework from which to interpret this data. The focus of this article is to describe the old framework and its' limitations, provide rationale for a new framework, and give examples of how this new framework can now be applied to clinical care.

## The old framework for interpreting inflammatory disorders

In science and medicine, a framework, or paradigm, is a theoretical or conceptual structure for defining and organizing information and relationships within a system. Rules and models within a framework are used to understand the relationship and interaction between the components, and these lead to predictions about processes and outcomes within the larger framework.

The paradigm for western medicine in the twentieth century is the germ theory of disease. The premise is that a single pathologic factor causes complex disorders. The germ theory was developed following technical advances of the compound microscope (allowing bacteria to be observed), culture and sterilization techniques (e.g., work of Lister and Pasture), epidemiologic evidence of infections causing disease (e.g., John Snow and the cholera epidemic in London coming from the Broad street pump), and the work of Koch to define the process of proving that an agent causes a disease (Koch's postulates).

Twentieth century Western medicine was built on the germ theory framework. Definitions of various diseases relied on tissue pathology which was expected to reveal the underlying infectious or parasitic agent causing inflammation or cancer. If there was inflammation without infection, then the disorder was defined by the type and duration of inflammation, with the expectation that research, using Koch's postulates, would eventually reveal the etiologic factor. From a clinical setting, combinations of signs and symptoms were used as surrogate markers of underlying pathology, and the idea of “functional” syndromes described clinical complaints when there was obvious tissue pathology. Thus, most medical disorders are classified by pathology rather than etiology, and this framework is the basis of modern disease taxonomy (e.g., ICD-9, ICD-10 codes).

Twentieth century biomedical research was also built on the germ theory framework. The scientific method taught in medical schools following the Flexner Report of 1910 (Flexner, 1910) was developed for identifying a *single* factor that caused a complex disease. The conceptual framework led to the process of rapidly evaluating a series of potential independent factors that were either included or excluded as the cause of disease based on *simple statistical tests* (null-hypothesis significance testing). The problem of experimental variance was addressed by increasing study size so that the effect of the primary etiologic factor within a *population* of subjects could be clearly identified. The result was a rapid progress in understanding, defining, and organizing infectious diseases, toxic agents, and Mendelian genetic traits. In each of these cases, a single factor was responsible for a complex disease syndrome.

The optimism of twentieth century Western medicine and the “scientific method” following the Flexner report diminished in the latter decades of the twentieth century when the simplistic approach failed to identify single etiologic factors for chronic inflammatory diseases, functional disorders, and cancers. Four examples of these failures have been highlighted elsewhere (Whitcomb, 2012), and are summarized here.

### Tissue is the issue

A major thrust of twentieth century Western medicine was the development and improvement of minimally invasive techniques to obtain tissue samples in living patients since this was the basis of disease diagnosis and treatment. Indeed, methods to obtain biopsies by endoscopic techniques, fine needle aspirates guided by CT, ultrasound and other techniques, laparoscopy and high-resolution imaging techniques were perfected. However, sophisticated methods of getting a tissue biopsy that were interpreted with early twentieth century criteria *did not* lead to significant improvement in medical management.

### Failed reproducibility

A second problem was identified when larger and more sophisticated clinical studies were conducted to define the etiology of more complex chronic diseases. The results of small and medium sized studies were often noted to be conflicting or non-reproducible. It was suspected that the epidemiological techniques that were used in many of the studies were flawed, and experimental design questions were raised. Evidence-based medicine (EBM) was added to the scientific approach to address these issues (Timmermans and Mauck, 2005). Among the many problems of EBM is the fact that it relies on data that was collected in previous trials that were designed based on theories that were often 15–20 years out of date. Furthermore, the strict criteria that are necessary for developing EBM guidelines were found to exclude large numbers of patients and those disorders that fell outside of the mean of the population without insight. And, depending on the available data and criteria, different groups who use EBM to develop guidelines often come to different conclusions. In reality, EBM is really more of a medical literacy exercise than a way to provide new insights into complex diseases (Wyer and Silva, 2009). EBM that remains within the germ-theory paradigm will primarily be of value in simple diseases, where it rarely provides any new insights.

### Minimal effects of common SNPs

There has been great hope that mapping, and then sequencing the human genome would identify the gene that causes “your-favorite-disease”. A common approach was the GWAS, which was developed to quickly identify the genetic variants causing a variety of disorders and diseases (Witte, 2010). The approach, however, was developed within the framework of the germ theory of disease, and the scientific method of null-hypothesis significance testing (i.e., the frequency of each genetic variant is compared between cases and controls using a simple chi square or exact test, with “significance” based on a study power calculation, adjusted for the number of other SNPs tested). However, it was discovered that complex diseases have *many* genetic variants that are statistically associated with disease, but they only have a very small effects, and the presence of absence of a SNP in a patient usually has no clinical relevance. Furthermore, to determine these small effect genetic variants required huge numbers of patients, with the expectation of a minimum of 1000 cases and 1000 controls (Ioannidis et al., 2001), and still suffers from false discovery (Benjamini and Hochberg, 1995). In more complex common diseases, tens of thousands of patients are being included in each arm of the study (Nettleton et al., 2010). However, the additional data is not bringing further insight into the disease in a way that provides clinically fashionable insights.

### Interpreting data without statistics

The final technological breakthrough is next generation sequencing (NGS). This technology has the potential of rapidly sequencing an individual's entire DNA sequence for a few thousand dollars. The problem with NGS is that hundreds to thousands of unexpected genetic variants are discovered in each person's DNA sequence, and it is impossible to demonstrate the effect of each variant based on statistical methods. Together, these technology breakthroughs illustrate the inadequacy of the twentieth century western medicine disease paradigm interpretation of complex disorders in a germ theory model.

## The new framework for twenty-first century medicine

The great frontier in CP is applied physiology. The new framework needed for medicine is based on integrative physiology, cell biology, systems modeling, and simulation of biological processes in individuals where multiple variables associated with various components of a system, or the external forces that influence them, are considered in individual patients (i.e., individualized or personalized medicine). The need to move away from research based on null-hypothesis significance testing and toward modeling is being recognized (Rodgers, 2010), but the current approaches of systems biology at a molecular level are likely unnecessary in disease modeling (Whitcomb, 2012). Furthermore, the germ theory of disease does not need to be abandoned. It needs to be placed in the context of the new framework as a situation where the number of variants resulting in disease equals one. A personalized medicine approach is needed when a syndrome is complex such that multiple etiologies or combination of factors lead to the same pathology, when the same pathology leads to multiple outcomes and/or when the results of interventions are unpredictable. Therefore, they are needed for chronic inflammatory diseases such as CP, functional disorders such as chronic pain in minimal change pancreatitis, and cancers including pancreatic cancer. Personalized medicine focuses on disease mechanism rather than association; it relies on modeling and simulation rather than classification, but it will be able to provide guidance for individuals rather than for subsets of a population.

## North american pancreatitis study 2 (NAPS2)

**Figure 1:**
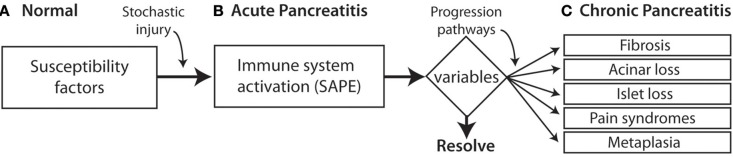
**SAPE progression model. (A)** Normal histology. Patients may have genetic risk factor and alcoholism but without pancreatic inflammation. **(B)** Acute pancreatitis is triggered by a stochastic injury (e.g., gallstone) leading to acute pancreatitis with activation of the innate immunes system a recruitment of inflammatory cells. A variety of modifying factors and variables (triangle) determine the resolution of acute pancreatitis, or contribute to a variety of pathways that lead to the recognized components of the chronic pancreatitis syndrome. **(C)** Chronic pancreatitis reflects irreversible damage manifests by the response of multiple cell types.

North American Pancreatitis Study 2 (NAPS2) is multicenter study that was designed by the author in the late 1990s in anticipation of future modeling in simulation approaches that might *prevent* CP (Whitcomb et al., 2008). Rather than using traditional classification approaches to CP the NAPS2 program took a broad view, envisioning pre-existing risk, stochastic events initiating an inflammatory process that was manifest clinically by episodes of recurrent acute pancreatitis or recurrent pain [i.e., the sentinel acute pancreatitis event (SAPE) hypothesis model (Whitcomb, 1999; Yadav and Whitcomb, 2010)]. Continuation and variations of inflammatory progresses then resulted in a constellation of variations in specialized cell and systems with dysfunctions recognized as of different clinical complications. Activation of pancreatic stellate cells leads to fibrosis. Acinar cell loss or dysfunction results in diminished digestive enzyme production with maldigestion. Islet cell dysfunction leads to endocrine failure with diabetes. Nerve injury and pathologic adaptation leads to chronic pain syndrome, and abnormal transition of inflamed pancreatic acinar-duct cells leads to pancreatic cancer (Figure 1).

Prior to NAPS2, there was no systematic way to classify susceptibility factors, other risk factors or combinations of factors. An etiologic-based classification system had to be invented which is known as the TIGAR-O system (Etemad and Whitcomb, 2001), which classifies factors as either Toxic-metabolic (e.g., alcohol, smoking), Idiopathic (e.g., tropical pancreatitis, early or late onset), Genetic, Autoimmune, Recurrent-acute or severe (e.g., 95% pancreatic necrosis in acute pancreatitis) or Obstructive. This is contrast to the definitions of the Marseille classification system that defines acute pancreatitis and CP by traditional clinical and pathologic criteria (Sarles, 1965; Singer et al., 1985) and the Cambridge classification system (Sarner and Cotton, 1984) which defines ages of progressive destruction but provides no insight into the mechanism of disease.

The rate of progression from first symptom to the diagnosis of CP or evidence of exocrine or endocrine failure and cancer was considered to be important. The NAPS2 questionnaires were designed to facilitate construction of timelines, with the dates of key events recorded so that the CP could be modeled as a disease process rather than a diagnosis, and the effect of interventions evaluated. This was put within the framework of the SAPE hypothesis in contrast to using a diagnosis ICD9 577.1 alone. By modeling pancreatitis as an evolving process, susceptibility factors and the types of stochastic events that initiate the process could be identified and quantified, and the role of an acute pancreatitis event and other variables that initiate and drive the progression to CP could be organized, measured, and studied in a series of individual patients. Thus, multiple variables could easily be classified as risk factors, biomarkers, endpoints, or surrogate endpoints and used for constructing predictive models which anticipated the development of complications and allow for etiology based treatments to prevent the progression of diseases before the symptoms develop. In addition, biological samples from consecutive patients were collected and processed for DNA and serum and/or plasma for biomarker studies.

The utility of this approach has been remarkable. It has allowed the North American Pancreatic Study Group to subdivide CP into etiology-based processes that all have the same clinical appearance and pathologic features. This allows for early recognition process and targeting the etiology rather than the symptoms. Much of the data from the first 1000 patients has now been published. Surprisingly, there appear to be a threshold for risk of alcoholic pancreatitis at five more drinks per day (60 ounces of alcohol per day), and only 15% of total patients of all patients drank at this level (Yadav et al., 2009; Cote et al., 2010). The majority of these patients were in the CP group and very few were in the recurrent acute pancreatitis group, suggesting that alcohol also caused rapid progression from recurrent acute to CP so that in a cross sectional study, the pancreatitis category was markedly enriched. Smoking was also found to be a strong, independent, and synergistic risk factor for CP which is often not recognized by general practitioners as well as experts in CP (Yadav et al., 2011). Genetic etiologies (*CFTR*, *SPINK1*, and *PRSS1*) contributed to about 25% of the total cases (Whitcomb, 2011). More interesting was that about 40% of patients were idiopathic. These are the ones for whom we believe complex and environmental factors play a more dominate role. We expect that GWAS will bring further insight into this category.

## Modeling chronic pancreatitis

**Figure 2:**
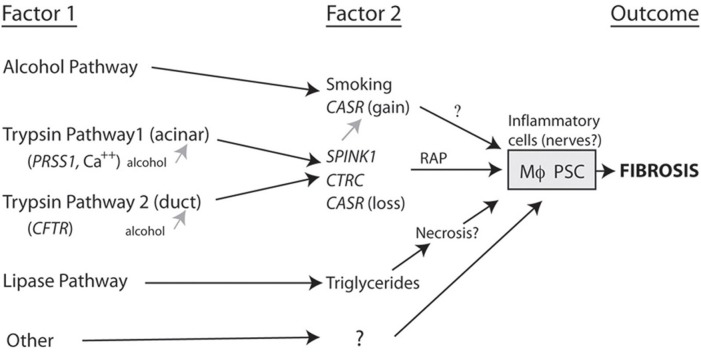
**Complex pathways to fibrosis.** A combination of two or more factors that together markedly increase the likelihood that fibrosis will develop. For fibrosis to occur the risk factor combination must converge on the inflammatory cells (e.g., macrophages, Mϕ; pancreatic stellate cells, PSC). Note that alcohol can increase susceptibility to pancreatitis by acting on the acinar cell and/or duct cell, but appears to drive CP through another *SPINK1*-independent pathway (Aoun et al., 2008).

The framework for beginning to build models of pancreatic disease includes classifying patients as combinations of factors that occur together, in distinction to factors that are only seen together by chance (Aoun et al., 2008). Figure 2 is a working model of etiology-based pathway in which two factors are required to drive the macrophages and pancreatic stellate cells to cause fibrosis.

As seen in the figure, alcohol plus a second factor markedly increases the risk of fibrosis. Trypsin-related pathways can begin either in the acinar cell or in the duct and these appear to converge with high risk being linked to secondary factors such as *SPINK1*. Lipase and lipid metabolism disorders may represent a separate pathway with liptoxicity or other factors directly causing pancreatic injury and CP as well as other mechanisms that are yet to be fully defined. These organization diagrams can set the stage for assigning relative effect strengths to various components of primary and secondary factors as well as other environmental and metabolic effects that may alter the rate of progression from the onset of pancreatitis to fibrosis. The most important of these to-be-named variables are genetic variants.

NGS is an enabling technology for rapidly revealing all variants in the genome of an individual patient. The complexity of the human genome has been recognized, and it is further recognized that it will be nearly impossible to evaluate variants of entire genome of an individual statistically. The challenges in analysis of NGS for most disorders, especially those with confounding environmental variables, are almost insurmountable. However, this is not true for the pancreas.

The advantage of developing methods for NGS analysis in the pancreas is that it is a very simple organ in which the exocrine pancreas has two primary cell types, the duct cell and the acinar cell. Each of the two cell types has a primary function (bicarbonate secretion or enzyme synthesis). The mechanism of injury is trypsin activation in most cases. The time of initial injury is often known, and the immune response is fairly stereotypic. Thus, for trypsin related susceptibility factors, five major genes have been identified that have been associated with patients with CP. In addition to *PRSS1*, *CFTR*, and *SPINK1*, the *CTRC* and *CASR* genes are additional risk factors that appear to increase risk of pancreatitis in the context of one of three primary susceptibility factors (Larusch and Whitcomb, 2011; Schneider et al., 2011; Rosendahl et al., 2012). As noted in Figure 2, the CTRC is envisioned to be linked to the trypsin pathway where as the CASR gain of function mutations are found in alcoholic patients, and CASR loss of function mutations are found in trypsin-associated pathways. The advantage of using NGS is that it is less expensive to use whole exome sequencing for the entire 30,000 + genes than it is to sequent CFTR using standard sanger sequencing technology. We already demonstrated the utility in a family with idiopathic HP (Larusch et al., 2012). This case demonstrated that four risk factors combined in different patients within the family tree in complex ways to cause pancreatitis from slightly different etiologies in each of the four affected individuals. This was done by focusing on the five known susceptibility genes rather than analyzing the entire human genome. What was amazing is that unexpected variants were found in these patients including a copy number variant of the *PRSS1* gene in one patient, a rare *SPINK1* mutation that had only been described in two French patients in early 2004 (Le Marechal et al., 2004), a strong effect of smoking, and a *CFTR* variant that is considered mild variable may be associated with pancreatitis disease (Larusch et al., 2012). This is a powerful proof of principle to illustrate a practical approach to the use of NGS for pancreatic disease. Of note, caution must be taken when evaluating *PRSS1* variants using NGS since disease-causing mutations are often gene conversion mutations from different forms of trypsin or trypsin pseudo-genes (Chen and Ferec, 2000) so that there is a high risk of variants in the trypsin genes being identified in these patients. Therefore, we always use very specific methods to confirm true mutations in the *PRSS1* genes when analyzing patients at risk for pancreatitis.

The final question is whether or not this new framework is compatible with clinical practice. At the University of Pittsburgh we have reorganized our pancreas clinic so that genetic testing occurs very early in the workup rather than at the end (Whitcomb, 2012). In our experience and in the NAPS2 study (unpublished, Whitcomb et al., 2012) we have found that there is often a delay of 6–10 years between the onset of symptoms and the development of CP to the point where a diagnosis could be made. The diagnosis of CP requires irreversible damage to the pancreas, which is exactly what we are trying to avoid! The 6 year delay does not mean that there is no disease; it means that the criteria of the old paradigm have not been met. Use of genetic testing identifies what part of normal physiology is likely to be disrupted by genetic variation and has implications for which cell type is likely to be the culprit in initiating the pathology. Therefore, attention to either the large ducts, the small pancreatic ducts, the acinar cell, or the immune system before the destruction of the pancreas allows targeted therapies to be initiated that address the etiology rather than covering the symptoms. We believe that this is a new paradigm for approaching complex inflammatory disease with late complications that should be avoided at all costs.

How can physicians receive and interpret the flood of information that is expected to come with whole genome sequencing and new “omics” biomarkers? The answer is that this is clearly impossible. What is needed is the development of decision support tools that are able to rapidly scan all of the information from an individual patients genome and biomarker studies, structure it in an organized way, perform calculations and simulations, and provide the physician with a few options, their likelihood of being successful, and how to best monitor the patient if interventions are made. The role of the physician will also be to use their own training and clinical experience to help guide the treatment of patients in whom there are no helpful predictions. Thus, there will be a lot to discover and apply in twenty-first century medicine.

## Summary

We believe that genetics will be the foundation of clinical management of pancreatic diseases in the future. New recognition that the development of CP is associated with a limited number of etiologies, and recognition that there may be several years between the first symptoms and organ destruction is a call to develop early and effective interventions that are based on the *etiology* rather than symptoms and complications. While the full spectrum of genetic variants that is linked to pancreatic disease or have not yet been described, the new framework that is necessary for their interpretation is already here.

### Conflict of interest statement

Dr. Whitcomb has served as a consultant for Abbott, Eli Lilly, Indianapolis, IN, USA, Millennium Pharmaceuticals, Cambridge, MA, USA and Novartis, Basal, Switzerland. He is Editor, Pancreatic Diseases for UpToDate, Waltham, MA, USA. He owns stock in Ambry Genetics and also the US patent 6406846 entitled “Method for determining whether a human patient is susceptible to hereditary pancreatitis, and primers therefore,” which has been licensed and provides royalty income.
